# Mixed Invasive Adenoid Cystic and Lobular Carcinoma of the Breast: A Case Report of a Rare Hybrid Tumor

**DOI:** 10.7759/cureus.101902

**Published:** 2026-01-20

**Authors:** Maha S Babker, Hector Chavarria

**Affiliations:** 1 Pathology and Laboratory Medicine, University of South Alabama Frederick P. Whiddon College of Medicine, Mobile, USA

**Keywords:** adenoid cystic carcinoma, breast cancer, breast histopathology, e-cadherin, hormone receptor positive, lobular carcinoma, mixed carcinoma, molecular pathology, rare breast cancer subtypes, triple-negative

## Abstract

Adenoid cystic carcinoma (ACC) of the breast is an exceedingly rare subtype of triple-negative breast cancer, accounting for less than 0.1% of all breast malignancies. In contrast, invasive lobular carcinoma (ILC) is a more common special histologic type, comprising 5-15% of breast cancers and typically characterized by estrogen receptor positivity and complete loss of epithelial cadherin (E-cadherin) expression. The coexistence of these two biologically and morphologically distinct neoplasms within the same breast lesion is exceptionally rare and, to our knowledge, has not been previously documented.

A 62-year-old Caucasian female patient presented with nonspecific stinging discomfort in the right breast. Diagnostic imaging revealed a 1.8 cm irregular, spiculated mass. A core needle biopsy demonstrated invasive mammary carcinoma that was estrogen receptor-positive, progesterone receptor-positive, and human epidermal growth factor receptor 2 (HER2)-negative. Following right breast mastectomy and sentinel lymph node biopsy, final pathology revealed a biphasic neoplasm composed of both ACC and lobular carcinoma (LC) components. The ACC component exhibited classic cribriform and tubular architecture, a triple-negative immunophenotype, strong transformation-related protein 63 (p63) and cluster of differentiation 117 (CD117; c-Kit) positivity, and a low Ki-67 proliferation index (9.8%). The LC component lacked a classic single-file growth pattern but showed dyscohesive cell clusters with complete loss of E-cadherin expression, strong estrogen receptor positivity (94.18%), and a markedly elevated Ki-67 index (42.9%). All seven sentinel lymph nodes were negative for metastatic carcinoma.

This rare case of synchronous ACC and ILC underscores the diagnostic complexity of composite breast tumors. Accurate classification requires careful correlation of histomorphology with immunohistochemical findings, particularly when one or both components exhibit non-classical architectural patterns. Given the contrasting biological behavior of triple-negative ACC and hormone receptor-positive ILC, an individualized, multidisciplinary management strategy is essential. Increased awareness of such mixed neoplasms may enhance diagnostic precision and improve patient care.

## Introduction

Adenoid cystic carcinoma (ACC) of the breast is an exceptionally uncommon malignancy, representing well under 0.1% of all breast cancers [1]. Although it shares key features with its salivary gland counterpart, including v-myb avian myeloblastosis viral oncogene homolog (MYB)-driven oncogenesis and a dual epithelial-myoepithelial architecture, breast ACC typically follows an indolent clinical course, with long-term survival rates exceeding 90%. Three histologic variants are recognized: the classic cribriform or tubular type, the solid-basaloid form, and high-grade transformed ACC, each associated with distinct biological behavior [2]. Most breast ACCs demonstrate a triple-negative (estrogen receptor (ER)-negative/progesterone receptor (PR)-negative/human epidermal growth factor receptor 2 (HER2)-negative) immunophenotype, yet they generally lack the aggressive course typical of other triple-negative breast cancers.

In contrast, invasive lobular carcinoma (ILC), first described by Stewart and Foote in 1941 [3], is far more common, accounting for 10-15% of invasive breast cancers. Its hallmark is loss of epithelial cadherin (E-cadherin) expression due to inactivation of cadherin-1 (CDH1), resulting in the characteristic single-file, discohesive growth pattern. ILCs are typically ER-positive and may present at a higher stage, with a propensity for multifocality, bilaterality, and late recurrence [4]. Molecularly, phosphatidylinositol-4,5-bisphosphate 3-kinase catalytic subunit alpha (PIK3CA) mutations are frequent. In contrast, alterations in tumor protein p53 (TP53), HER2, phosphatase and tensin homolog (PTEN), and forkhead box A1 (FOXA1) occur less commonly but carry therapeutic relevance [5].

To our knowledge, synchronous ACC and lobular carcinoma (LC) arising within the same breast lesion have not been previously reported. The coexistence of these two tumors - each with distinct cellular origins, molecular drivers, receptor profiles, and expected clinical behavior - creates a unique diagnostic and therapeutic challenge. Documenting this mixed carcinoma expands the recognized morphologic spectrum of breast neoplasia, highlights diagnostic pitfalls when classic ILC architecture is lacking despite E-cadherin loss, and underscores management considerations when a triple-negative, MYB-driven component coexists with an ER-positive, CDH1-deficient counterpart.

## Case presentation

**Figure 1:**
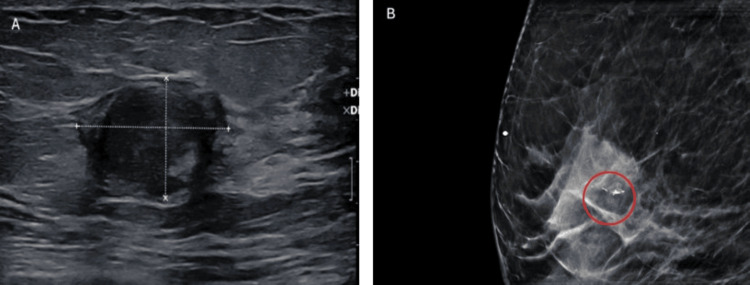
Preoperative imaging studies (A) Ultrasound image demonstrating a hypoechoic, irregularly shaped mass with indistinct margins, suggestive of a suspicious lesion, and (B) Mammogram showing a high-density mass associated with pleomorphic microcalcifications.

A 62-year-old Caucasian female patient presented with a six-month history of intermittent stinging discomfort in the right breast, without an associated palpable mass. Her past medical history was notable for a benign breast mass excised 10 years earlier, atrial fibrillation, and ulcerative esophagitis. She had no family history of breast cancer and reported a 40-pack-year cigarette smoking history. Diagnostic imaging with mammography and ultrasound demonstrated scattered areas of fibroglandular density. In the right upper mid-breast, an irregular, spiculated, high-density mass measuring approximately 1.8 cm with fine pleomorphic calcifications was identified, showing an interval increase compared with prior imaging (Figure 1).

**Figure 2:**
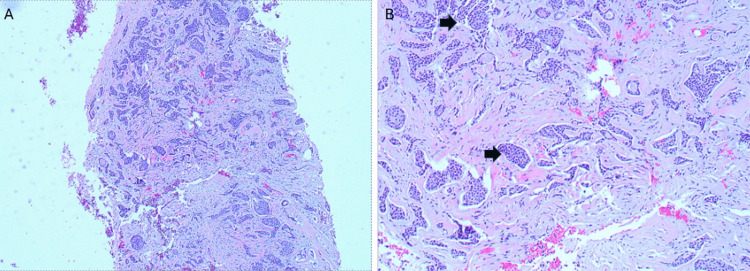
Needle biopsy (A) Core biopsy and (B) Hematoxylin and eosin (H&E)-stained core needle biopsy, demonstrating nonspecific histologic features consistent with invasive mammary carcinoma of no special type (NST). The arrows highlight a cluster of malignant epithelial cells within the stroma.

A core needle biopsy revealed an invasive mammary carcinoma of no special type (NST), grade two of three, with a combined histologic grade of six of nine (tubules 3/3, nuclear pleomorphism 2/3, mitotic activity 1/3). Due to limited biopsy tissue, immunohistochemical (IHC) stains to differentiate ILC from invasive ductal carcinoma were not performed. Biomarker testing demonstrated ER-positive, PR-positive, and HER2-negative (score zero) status. Representative histology from the biopsy is shown in Figure 2. 

**Figure 3:**
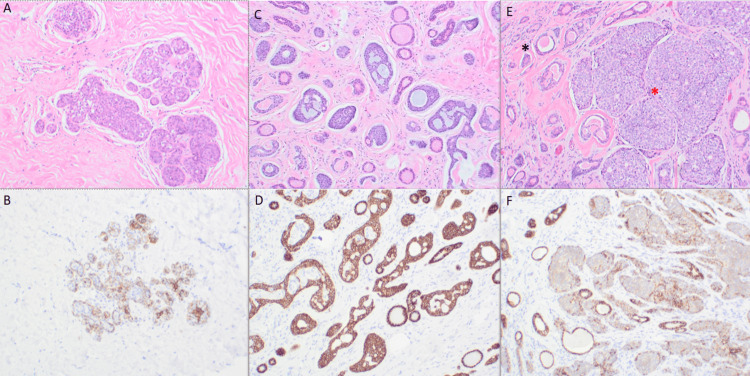
Immunohistochemistry (IHC) images (A) Hematoxylin and eosin (H&E) staining showing lobular carcinoma in situ (LCIS); (B) E-cadherin IHC demonstrating loss of expression in LCIS; (C) H&E showing adenoid cystic carcinoma (ACC); (D) E-cadherin IHC showing retained expression in ACC; (E) H&E highlighting pure ACC (black asterisk) and proliferating lobular carcinoma (red asterisk); (F) E-cadherin IHC confirming retained expression in ACC and loss in lobular carcinoma; small discohesive cells are interspersed within the ACC component.

The patient subsequently underwent a right mastectomy with sentinel lymph node biopsy. Final pathology revealed a biphasic invasive carcinoma composed of ACC and ILC, measuring 1.8 × 1.5 cm, with a Nottingham score of six of nine (3 + 2 + 1), grade 2 [6]. The tumor was located 2.8 cm from the posterior margin. Classic-type lobular carcinoma in situ (LCIS) was also identified. All seven sentinel lymph nodes were negative for metastasis. Low-power and high-power sections confirming the biphasic morphology are shown in Figure 3.

**Figure 4:**
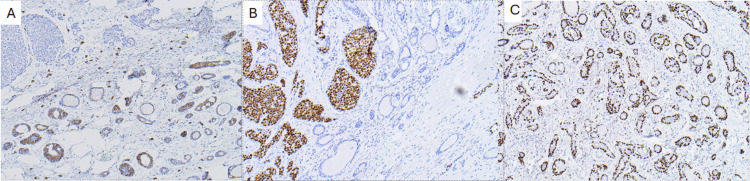
Immunohistochemical (IHC) staining findings (A) Cluster of differentiation 117 (CD117; c-Kit): Cytoplasmic positivity in the epithelial component of adenoid cystic carcinoma (ACC), aiding in its identification; (B) GATA binding protein 3 (GATA3): Diffuse nuclear positivity in lobular carcinoma components; (C) Transformation-related protein 63 (p63): Strong nuclear staining in myoepithelial/basal cells, highlighting the myoepithelial component of ACC.

Histologically, the ACC component demonstrated classic cribriform and tubular architecture without perineural or lymphovascular invasion. IHC for the ACC component showed positivity for p63, cluster of differentiation 117 (CD117; c-Kit), E-cadherin, focal S100 protein, and patchy smooth muscle myosin heavy chain (SMMHC). It was negative for GATA binding protein 3 (GATA3), calponin, and mammaglobin, as shown in Figure 4.

The LC component consisted of dyscohesive epithelial cells lacking E-cadherin expression. Although the typical single-file infiltration pattern was absent, the LC cells showed loss of cellular cohesion and were intimately admixed with the ACC component. IHC for the ILC component demonstrated strong nuclear GATA3 positivity and mammaglobin expression, with S100 and SMMHC patterns, similar to those of the ACC component. The LC component was strongly ER-positive (94.18%), PR-negative, and had an elevated Ki-67 proliferation index of 42.9%. In contrast, the ACC component was ER-negative, PR-negative, and HER2-negative with a Ki-67 index of 9.8%, consistent with a triple-negative phenotype. HER2/neu status assessed by in situ hybridization was negative. Representative IHC stains for both tumor components are shown in Figure 4.

## Discussion

ACC was first described as “cylindrical” carcinoma by Billroth in 1856, and the first reported case of breast ACC was reported by Geschickter in 1945. Although ACC most commonly arises in the salivary glands, it may also occur in the nasopharynx, trachea, uterine cervix, skin, lungs, kidneys, and breast. Compared to other anatomic sites, breast ACC carries a notably favorable prognosis. Despite its triple-negative immunophenotype [7], breast ACC typically exhibits indolent behavior and favorable outcomes, with an incidence of less than 0.1%. Reported relative survival rates at five, 10, and 15 years are 98.1%, 94.9%, and 91.4%, respectively [2]. Breast ACC most frequently occurs in the superolateral quadrant or beneath the areola, and approximately 14% of patients experience localized pain, likely due to perineural invasion and myoepithelial contraction as described by Kashiwagi et al. [2].

Breast ACC exhibits a morphologic spectrum similar to that of its salivary gland counterpart. Based on the architectural and cytologic features, three subtypes are recognized: classic ACC, solid-basaloid adenoid cystic carcinoma (SB-ACC), and ACC with high-grade transformation. The classic form demonstrates dual epithelial and myoepithelial populations arranged in cribriform and tubular structures, with characteristic pseudolumina containing basement membrane material. Immunohistochemically, the epithelial cells stain for cytokeratin 7 (CK7) and epithelial membrane antigen (EMA), whereas the myoepithelial component expresses transformation-related protein 63 (p63), S100 protein (S100), cytokeratin 5/6 (CK5/6), and calponin. Solid-basaloid adenoid cystic carcinoma and ACC with high-grade transformation represent the more aggressive variants. Areas of high-grade transformation may resemble high-grade ductal carcinoma, and molecular studies suggest these arise clonally from classic ACC, representing tumor progression.

At the molecular level, ACC is characterized by the recurrent translocation t(6;9)(q22-23;p23-24), which results in a MYB-NFIB fusion gene, the primary oncogenic mechanism of this tumor. MYB, a proto-oncogene located at chromosome 6q22 23, functions as a transcriptional regulator with strong oncogenic potential and is expressed in several types of malignant tumors. NFIB (nuclear factor I-B), located at chromosome 9p23 24, encodes a transcription factor involved in cell proliferation, apoptosis, and development. The MYB-NFIB fusion is not site-specific, indicating that this genetic alteration occurs across ACCs from different anatomic origins. Detection rates of the fusion in breast ACC vary depending on tissue preservation and the analytical platform. Brill et al. reported higher detection rates in frozen samples compared to paraffin-embedded tissue, with positivity observed in both primary and metastatic lesions [8].

ILC is the most common special histologic type of breast cancer. The term “lobular carcinoma” was introduced by Stewart and Foote in 1941 [3], who first emphasized loss of cell cohesion as the defining histologic hallmark of LCIS. ILC is characterized by dyscohesive, single-file infiltration of E-cadherin-negative tumor cells. Initially, LCIS was considered a prerequisite for the diagnosis of ILC; however, subsequent studies established that ILC may occur independently, particularly in elderly patients with atrophic breast parenchyma lacking distinct lobular structures. Today, ILC accounts for 10-15% of breast cancers and coexists with LCIS in only about half of cases. Foote and Stewart also recognized that tumors composed of loosely arranged cells likely have a lobular origin, even in the absence of identifiable LCIS, a concept later confirmed in male and experimental models lacking lobules [3].

Morphologically, ILC encompasses a broad spectrum, including classical, alveolar, trabecular, signet-ring cell-rich, histiocytoid, dispersed, and solid variants, with occasional apocrine differentiation or linear growth patterns. Despite this variation, loss of E-cadherin expression remains consistent across all types, confirming lobular differentiation [5]. Clinically, ILC tends to present in older women, often at higher pathological tumor stage (pT) and nodal stage but lower histologic grade compared with ductal carcinoma. It is also more frequently bilateral and overrepresented among de novo metastatic cases [5].

At the molecular level, ILC is characterized by CDH1 inactivation, typically resulting from frameshift mutations and 16q deletions, leading to loss of functional E-cadherin and disruption of the cadherin-catenin complex. This alteration affects β-catenin and p120-catenin signaling, promoting discohesion and infiltration. Other recurrent mutations include PIK3CA (30-50%), while TP53, HER2, PTEN, and FOXA1 mutations occur less frequently but are biologically relevant. Chromosomal imbalances are relatively rare, primarily involving gains at 1q/8p and losses at 16q, affecting hormone signaling and cell-growth pathways. Although typically classified as luminal A by gene expression assays (e.g., PAM50, Oncotype DX), a subset of proliferative molecular subtypes is associated with poorer outcomes, revealing a disconnect between histologic and molecular risk prediction [4,5,9].

In our case, the presence of the classical lobular in situ pattern and the apparent proliferation of LC cells within the ACC matrix suggest a possible phenomenon of clonal evolution or tumor hybridization. The diagnostic challenge lies in recognizing both components morphologically and confirming them immunohistochemically, particularly the loss of E-cadherin in the lobular component, which distinguishes it from ductal carcinoma. Given the divergent biology of triple-negative ACC and hormone receptor-positive ILC, a multidisciplinary, tailored therapeutic strategy is required. Due to the rarity of breast ACC, there is no established consensus regarding optimal management. Reported surgical options range from local excision to mastectomy [10]. Local excision carries recurrence rates of 6-37%, whereas recurrence is rare after mastectomy, leading many clinicians to favor mastectomy for definitive management [10,11]. Our patient underwent surgical resection followed by endocrine therapy with anastrozole. Systemic management was guided primarily by the hormone receptor-positive ILC component, while recognizing that the ACC component was triple-negative and is typically associated with an indolent clinical course.

## Conclusions

This case adds a rare hybrid entity of coexisting invasive ACC and ILC to the expanding spectrum of mixed breast tumors. It underscores the importance of meticulous histomorphologic evaluation, supported by adjunctive IHC, particularly p63, CD117; c-Kit, and E-cadherin, and careful integration with clinical and radiologic findings to achieve an accurate classification. Although both tumor components were small and node-negative, their divergent biology required individualized management, including surgical excision with negative margins followed by endocrine therapy targeting the hormone receptor-positive LC component, while acknowledging the generally indolent clinical behavior of the ACC component.

The absence of standardized guidelines for the management of breast ACC, combined with the extreme rarity of invasive ACC-ILC collision tumors, highlights the need for multi-institutional data collection and comprehensive molecular profiling to clarify tumor clonality, evolutionary pathways, and optimal adjuvant treatment strategies. Greater awareness of such hybrid histologies can improve diagnostic accuracy and better inform therapeutic decision-making that reflects the unique composite nature of these rare malignancies.
